# Metric basis and metric dimension of 1-pentagonal carbon nanocone networks

**DOI:** 10.1038/s41598-020-76516-1

**Published:** 2020-11-12

**Authors:** Zafar Hussain, Mobeen Munir, Ashfaq Ahmad, Maqbool Chaudhary, Junaid Alam Khan, Imtiaz Ahmed

**Affiliations:** 1 Department of Mathematics and Statistics, The University of Lahore, Lahore, 54000 Pakistan; 2 Department of Mathematics, University of the Punjab, New Campus Lahore, University of the Punjab, Lahore, 54590 Pakistan; 3 Department of Computer Science, COMSATS University Islamabad, Lahore campus, Defense Road Off Raiwind Road Lahore P. O. Box 54000, Pakistan, COMSATS University Islamabad, Lahore, 54000 Pakistan; 4 Department of Mathematics, IBA, Karachi, 75270 Pakistan; 5 University of Management and Technology, Lahore, 54000 Pakistan

**Keywords:** Computational chemistry, Applied mathematics

## Abstract

Resolving set and metric basis has become an integral part in combinatorial chemistry and molecular topology. It has a lot of applications in computer, chemistry, pharmacy and mathematical disciplines. A subset S of the vertex set V of a connected graph G resolves G if all vertices of G have different representations with respect to S. A metric basis for G is a resolving set having minimum cardinal number and this cardinal number is called the metric dimension of G. In present work, we find a metric basis and also metric dimension of 1-pentagonal carbon nanocones. We conclude that only three vertices are minimal requirement for the unique identification of all vertices in this network.

## Introduction

Metric Graph theory attempts to formulate the behavior of distance-based real-world systems. It has applications in chemistry, molecular topology, industrial chemistry and computer science^1,2^. It attracts people even from mathematics due to the interesting problems that originate from the structures and their symmetries involved. In an enigmatic network, it is always beneficial to uniquely recognize the position of nodes by giving an identity with respect to a special set. Such a set having minimum cardinal number is called a metric basis and its cardinal number is called the metric dimension^3,4^. In drug patterns these results have been used excellently to access particular atom.

Other significant applications of resolving sets and metric dimension can be traced in computer network, robot navigation, game theory and signal processing where largely a moving observer in a network system may be located by finding the distance from the point to the collection of sonar stations, which have been properly positioned in the network^2^. It is often desired to find exact location of a robot using resolving vector and this problem is well-known as robot navigation already has been studied in^1,2,4^. This particular set of vertices having minimum elements is called a resolving set of the graph space and the cardinal number of this set is called the metric dimension^5–7^. To choose minimal set of points is desirable to reduce the cost and time lapses.

The purpose of present research work is to establish the metric dimension and metric basis of the 1-pentagonal carbon nanocones networks. A fundamental problem in combinatorial chemistry is to represent mathematically the set of atoms, molecules and compounds in unique manner, in an enormous structure. In this way the vertices and edges of a labeled graph represent the atom and bond types respectively. Therefore, it elaborates non parallel mathematical representations for the vertices of a graph in such a way that different vertices have different representations by interpreting the graph of the structure under discussion^4,8–12^. Along with it, a 2D planar graph of 1-pentagonmal carbon nanocone is constructed in which atoms represent nodes and bonds represent the edges between them. For the fundamentals of graph theory, we refer to^12^. We consider hydrogen depleted molecular graphs in most of the cases.

**Figure 1:**
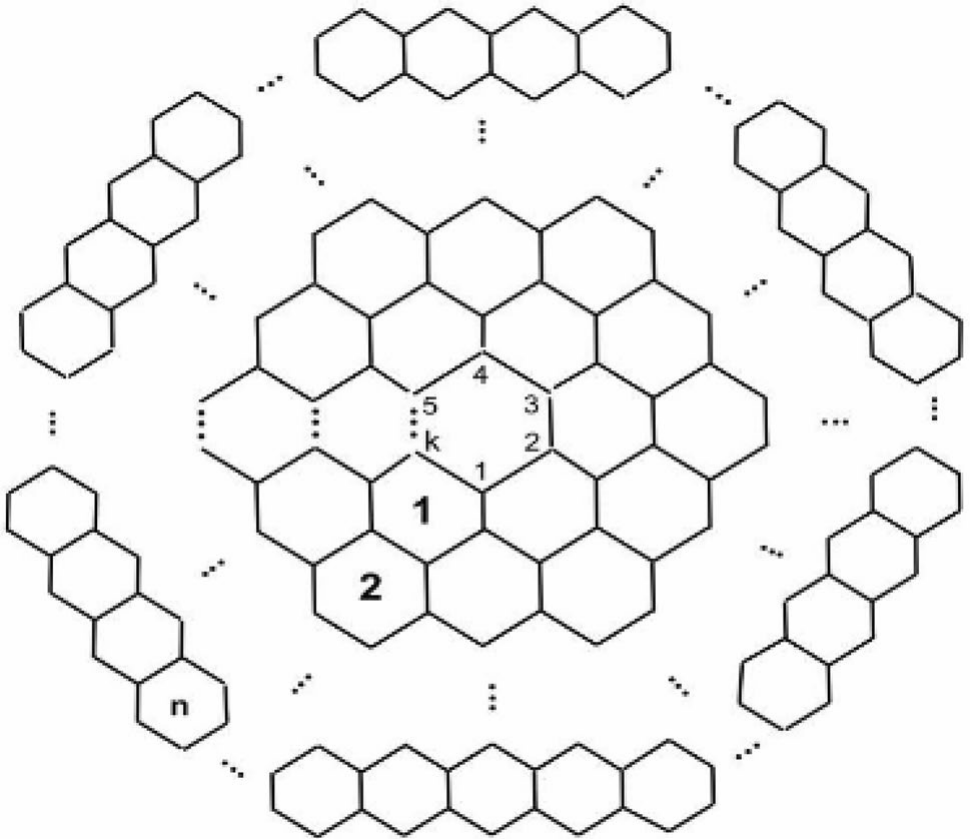
The molecular graph of carbon nanocones.

First noticeable appearance of Carbon nanocones came on the scene in 1968 or even before^13^, on the surface of naturally occurring graphite. These structures are fascinating because of their potential uses in energy storage, gas storage, gas sensors, biosensors, nano-electronic devices and chemical probes^14–16^. Nanocones are carbon networks that can be modeled as infinite cubic planar graphs. Authors in^14^ discussed mainly helical microtubules of graphitic carbon. Significant presence of carbon nanocones and combinatorial properties were discussed in^17,18^. Klein et al. classified carbon nanocones into eight classes on the basis of positive signed curvature^13^. Further classification and discussed of these structures have been elaborated in^15^ by Brinkmann et al.. Justus et al. discussed expander constants and boundaries of triangle patches of these nanocones^16^. More recently, carbon nanocones have gained increased scientific interest due to their unique properties and promising uses in many novel applications such as energy and hydrogen-storage^15^. The molecular graph of $CNC_{k} [n]$ nanocones consists of conical structures with a cycle of length $k$ at its core and $n$ layers of hexagons placed at the conical surface around its center, as shown in the following Fig. 1 which is molecular graph of carbon nanocones.

**Figure 2:**
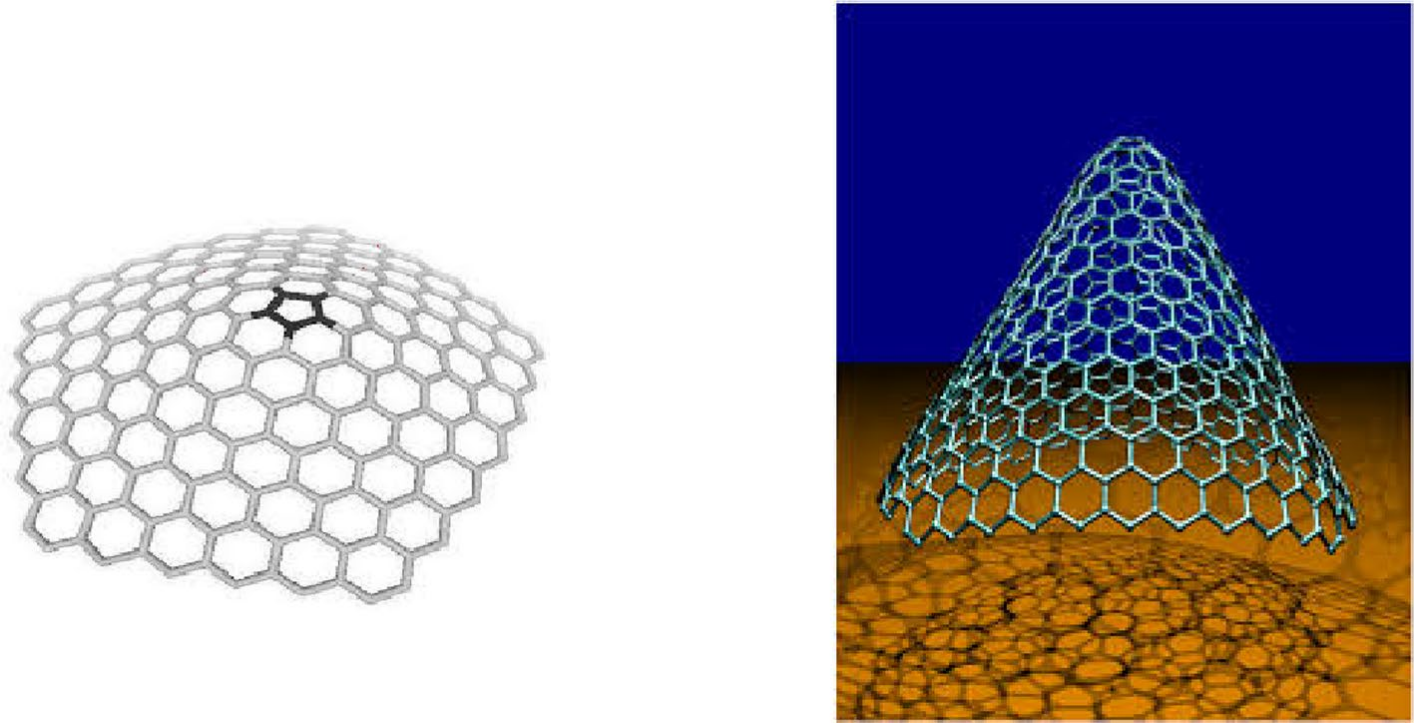
Carbon Nanocone $$CNC_{k} \left[ 5 \right]$$.

We are particularly interested in the case when $k = 5$ for the Fig. 1. A $k$-dimensional one-pentagonal nanocone is usually denoted by $CNC_{k} \left[ 5 \right]$ where $k$ is the number of hexagons layers encompassing the conical surface of the nanocone and 5 denotes that there is a pentagon on the tip called its core. Our notation is slightly different from one used in the above figure. The following Fig. 2 is actual picture of $CNC_{k} \left[ 5 \right]$^14,15,17,18^. Central single pentagon is shaded in black.

**Figure 3:**
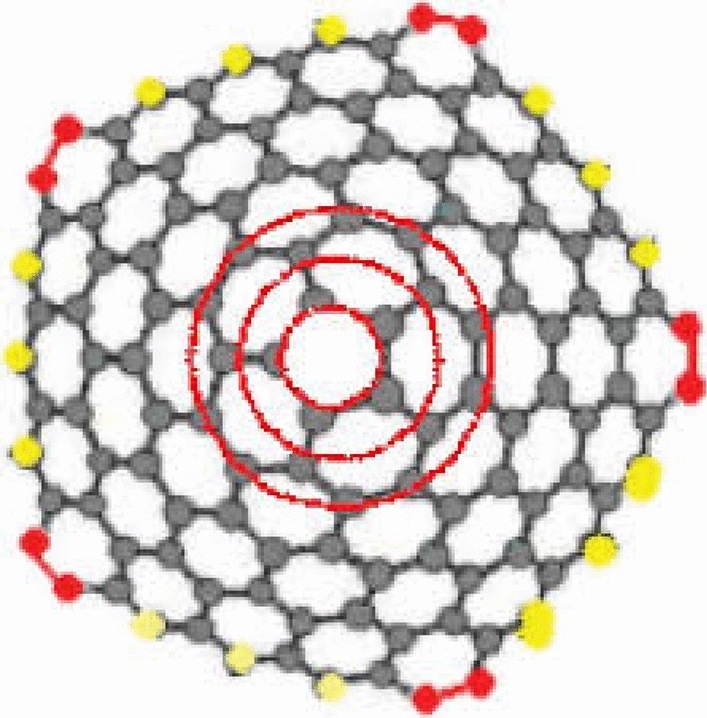
The Molecular graph of $CNC_{k} [5]$.

We transform the above structure into its molecular graph. Figure 3 represents molecular graph of $CNC_{k} [5][5]$.

Since we obtain a planar, simple and connected graph so we need to give overview of some basic facts in graph theory, see^3,12^. The distance $d(v_{1} ,v_{2} )$ between two vertices $v_{1} ,v_{2}$ of a connected graph $G$ is the length of the shortest path between $v_{1} {\text{ and }}v_{2}$ and is equal to the number of edges between $v_{1} {\text{ and }}v_{2}$ in the shortest path. For an ordered set of vertices $S = \{ w_{1} ,w_{2} ,w_{3} ,...w_{n} \}$, the n-vector $r(v|S) = (d(v,w_{1} ),d(v,w_{2} ),d(v,w_{3} ),...d(v,w_{n} )$ is called the vector representation $r(v|S)$ of $v$ with respect to $S$.The set $S$ is said to resolve the graph $G$ if the vector representations of all vertices of $G$ with respect to $S$ are distinct (see^1–7,10–13,16,19–21^). Inspired by the concepts used for dimensions, the idea of a general dimension for metric spaces was initialized by Blumenthal way back in 1953^22^. But because of continuous nature of Standard Euclidean space, its applications were few and far between. Consequently, not much was achieved in this context until Harary and Melter in 1975 specialized this idea for graphs with distance metric^3^. Today extensive literature exists for the dimensions of metric spaces of graphs as compared to a general metric space. A basis for $G$ is a resolving set with minimum cardinal number. The cardinal number of minimal resolving set is called the metric dimension of G, denoted by dim(G). Slater transformed these concepts of metric dimension and resolving sets for graphs^4^ and later on Melter and Harary independently explored these ideas in^3^. Resolving sets have been analyzed a lot since then. The concepts of resolving sets have been applied in a lot of fields including network discovery and verification^1,2,8,22,23^, connected joins in graphs, strategies in mastermind games^6^. A graph is said to be of constant metric dimension if the value of metric dimension does not alter with the choice of vertices. In^24^, authors discussed metric dimension of some graphs and proved that it is constant 1 if and only if graph is the path $P_{n}$. The metric dimension of complete graph $K_{n}$ is $n - 1$ for $n > 1$ and the metric dimension of cycle graph $C_{n}$ is 2 for n > 1^7^. In^20^, authors computed the metric dimension of the generalized Peterson graph. Some new results about the constant metric dimension have been discussed in^24^. Ali et al. computed partial results about the metrics dimension of classical Mobius Ladders in^8^, but correct and complete results are provided by Munir et al. in^9^. Recently in^10^, Zafar et al. proved that metric dimension of the alpha Boron nanotube depends upon the dimensions of the sheet. In^11^, authors computed not only the metric dimension of a generalized wheel graph ant-web gear graph but also gave an example of convex polytope with unbounded metric dimension. Recently authors in^19^ computed metric dimension of some families of some levels of Gear graphs. In^25^, the authors computed the metric dimension of circulant graphs. In^26^, authors computed explicit formula for the metric dimension of a regular bipartite graph. Authors discussed the metric dimension of the circulant and Harary graph in^27^.

In the present article we compute the classes of metric generator and metric dimension of $CNC_{k} [5]$. This subclass of carbon nanocones have been extensively studied recently. In^28^, authors discussed topological modeling techniques to the study one pentagon carbon nanocones and derived important results about preferred sizes and chemical reactivity. In^28^ authors also discussed the topological efficiency and topological roundness of $CNC_{k} [5]$ as the long-range topological potential whose local minima correspond to magic sizes of the nanocone with high probability of formation. In^29^ authors computed analytic expression of Hosoya polynomial and some distance-based indices like hyper Weiner and Hararay indices for 1-pentagonag carbon nanocones. In^30^ authors gave adjacent eccentric distance sum index of $CNC_{k} [5]$. An exact formula for the Wiener index of such nanocones is given in^31^ which is $\frac{62}{3}k^{5} + \frac{310}{3}k^{4} + \frac{1205}{6}k^{3} + \frac{1135}{6}k^{2} + 86k + 15$. In^32^ authors computed Pi and Szeged indices of one-pentagonal carbon nanocones. In^21^ authors computed closed analytic forms of the vertex Pi, szeged and omega polynomials of carbon nanocones. Recently in^33^ authors computed M-polynomial and some degree-based descriptors of molecular graphs of carbon nanocones.. Authors discussed some topological aspects of the line graph of carbon nanocone in^34^ using the technique of M-polynomial. In^35^ authors computed eccentricity connectivity index to be $5(10k^{3} + \frac{43}{2}k^{2} + \frac{43}{2}k + 4)$. We are interested in the metric dimension and metric basis of $CNC_{k} [5]$.

## Main results

In this section, we give our results. First result gives a sharp upper bound for $CNC_{k} [5]$. Next result computes sharp lower bound.

### Theorem 1

*For all *$k \ge 1$* we have *$\dim (CNC_{k} [5]) \le 3$*.*

### Proof

**Figure 4:**
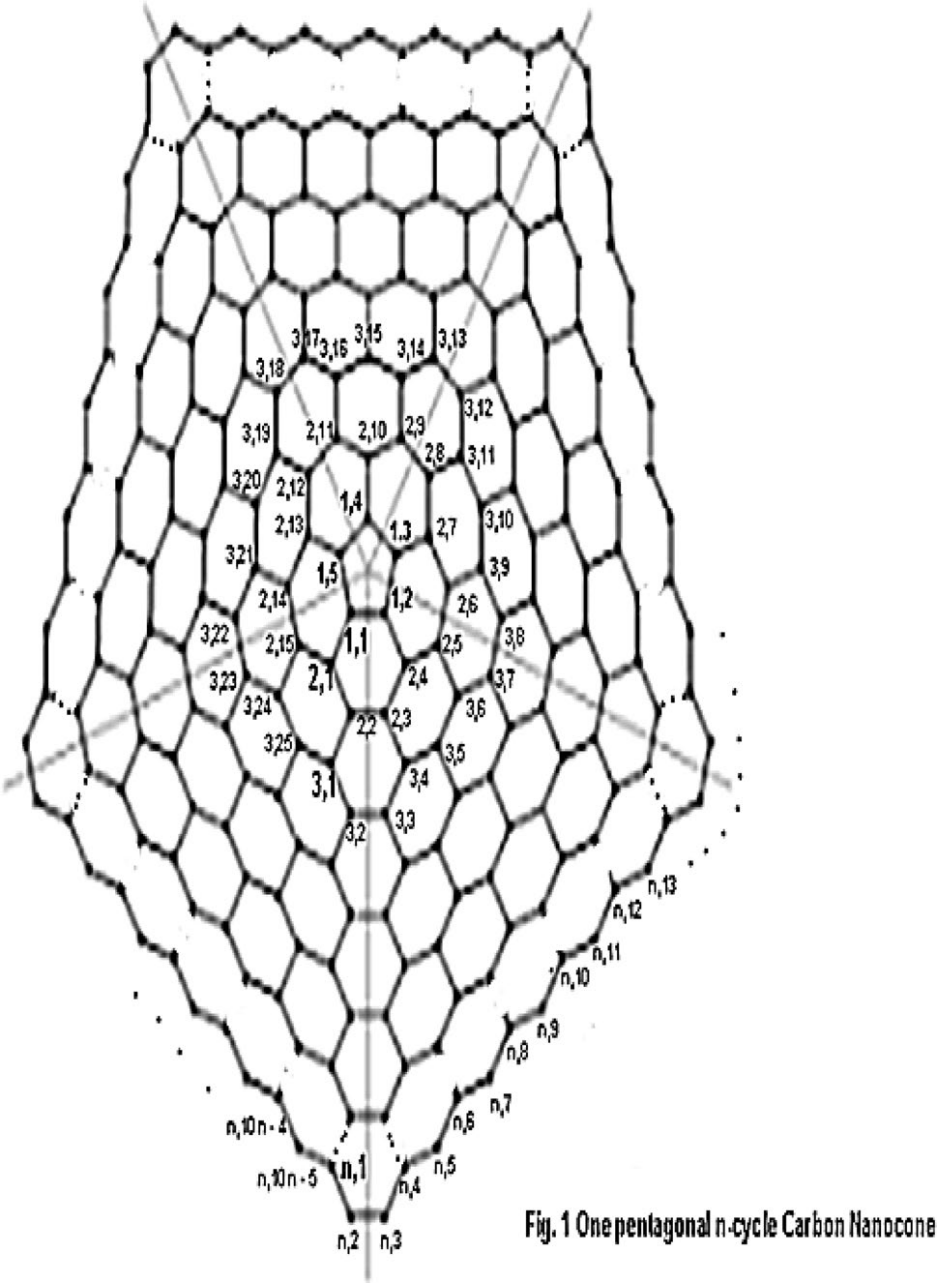
Labelling of 1-pentagonal Carbon Nanocone network.

The central cycle is a pentagon. For the rest of this article, we represent 1-petagonal Nano cones by $CNC_{k} [5]$. As the choice of efficient vertices lies in the heart of solution so we label the vertices in the attached Fig. 4. The vertices on ith cycle are { $v_{i,1} ,v_{i,2} ,v_{i,3} ,......,v_{i,10i - 5}$} where $1 \le i \le n$. The Fig. 4 below shows the graph of one pentagonal Carbon Nano cone network. We give the following labelling of the vertices of.

The vertex set of $$G$$ is partitioned as $$\{ v_{1,1} ,v_{1,2} ,v_{1,3} ,v_{1,4} ,v_{1,5} \} \cup$$ $$\{ v_{2,1} ,v_{2,2} ,v_{2,3} ,......,v_{2,15} \} \cup$$$$\{ v_{3,1} ,v_{3,2} ,v_{3,3} ,......,v_{3,25} \} \cup$$ $$\{ v_{4,1} ,v_{4,2} ,v_{4,3} ,......,v_{4,35} \} \cup$$ $$\cup \{ v_{i,1} ,v_{i,2} ,v_{i,3} ,......,v_{i,10i - 5} \} \cup$$ $$\cup \{ v_{n,1} ,v_{n,2} ,v_{n,3} ,......,v_{n,10n - 5} \}$$.

Let $W = \{ v_{n,1} ,v_{n,3} ,v_{n,2n + 1} \}$. We shall prove that W is a resolving set for $G$. The vector representations of vertices lying on first cycle are $r(v_{1,1} |W) = (2n - 3,2n - 1,2n - 1)$, $r(v_{1,2} |W) = (2n - 2,2n - 2,2n - 2)$, $r(v_{1,3} |W) = (2n - 1,2n - 1,2n - 1)$, $r(v_{1,4} |W) = (2n - 1,2n,2n)$, $r(v_{1,5} |W) = (2n - 2,2n,2n)$.

Representations of vertices on second cycle are $r(v_{2,1} |W) = (2n - 4,2n - 2,2n)$,$r(v_{2,2} |W) = (2n - 5,2n - 3,2n - 1)$, $r(v_{2,3} |W) = (2n - 4,2n - 4,2n - 2)$, $r(v_{2,4} |W) = (2n - 3,2n - 3,2n - 3)$, $r(v_{2,5} |W) = (2n - 2,2n - 2,2n - 4)$, $r(v_{2,6} |W) = (2n - 1,2n - 1,2n - 3)$, $r(v_{2,7} |W) = (2n,2n,2n - 2)$, $r(v_{2,8} |W) = (2n + 1,2n + 1,2n - 1)$, $r(v_{2,9} |W) = (2n + 1,2n + 2,2n)$, $r(v_{2,10} |W) = (2n,2n + 1,2n + 1)$, $r(v_{2,11} |W) = (2n + 1,2n + 2,2n + 2)$, $r(v_{2,12} |W) = (2n,2n + 2,2n + 2)$, $r(v_{2,13} |W) = (2n - 1,2n + 1,2n + 1)$, $r(v_{2,14} |W) = (2n - 2,2n,2n + 2)$, $r(v_{2,15} |W) = (2n - 3,2n - 1,2n + 1)$.

Representations of vertices on third cycle are $r(v_{3,1} |W) = (2n - 6,2n - 4,2n)$, $r(v_{3,2} |W) = (2n - 7,2n - 5,2n - 1)$, $r(v_{3,3} |W) = (2n - 6,2n - 6,2n - 2)$, $r(v_{3,4} |W) = (2n - 5,2n - 5,2n - 3)$, $r(v_{3,5} |W) = (2n - 4,2n - 4,2n - 4)$, $r(v_{3,6} |W) = (2n - 3,2n - 3,2n - 5)$, $r(v_{3,7} |W) = (2n - 2,2n - 2,2n - 6)$, $r(v_{3,8} |W) = (2n - 1,2n - 1,2n - 5)$, $r(v_{3,9} |W) = (2n,2n,2n - 4)$, $r(v_{3,10} |W) = (2n + 1,2n + 1,2n - 3)$, $r(v_{3,11} |W) = (2n + 2,2n + 2,2n - 2)$, $r(v_{3,12} |W) = (2n + 3,2n + 3,2n - 1)$, $r(v_{3,13} |W) = (2n + 3,2n + 4,2n)$, $r(v_{3,14} |W) = (2n + 2,2n + 3,2n + 1)$, $r(v_{3,15} |W) = (2n + 3,2n + 4,2n + 2)$, $r(v_{3,16} |W) = (2n + 2,2n + 3,2n + 3)$, $r(v_{3,17} |W) = (2n + 3,2n + 4,2n + 4)$, $r(v_{3,18} |W) = (2n + 2,2n + 4,2n + 4)$, $r(v_{3,19} |W) = (2n + 1,2n + 3,2n + 3)$, $r(v_{3,20} |W) = (2n,2n + 2,2n + 4)$, $r(v_{3,21} |W) = (2n - 1,2n + 1,2n + 3)$, $r(v_{3,22} |W) = (2n - 2,2n,2n + 4)$, $r(v_{3,23} |W) = (2n - 3,2n - 1,2n + 3)$, $r(v_{3,24} |W) = (2n - 4,2n - 2,2n + 2)$, $r(v_{3,25} |W) = (2n - 5,2n - 3,2n + 1)$.

Representations of vertices on 4th cycle are $r(v_{4,1} |W) = (2n - 8,2n - 6,2n)$, $r(v_{4,2} |W) = (2n - 9,2n - 7,2n - 1)$, $r(v_{4,3} |W) = (2n - 8,2n - 8,2n - 2)$, $r(v_{4,4} |W) = (2n - 7,2n - 7,2n - 3)$, $r(v_{4,5} |W) = (2n - 6,2n - 6,2n - 4)$, $r(v_{4,6} |W) = (2n - 5,2n - 5,2n - 5)$, $r(v_{4,7} |W) = (2n - 4,2n - 4,2n - 6)$, $r(v_{4,8} |W) = (2n - 3,2n - 3,2n - 7)$, $r(v_{4,9} |W) = (2n - 2,2n - 2,2n - 8)$, $r(v_{4,10} |W) = (2n - 1,2n - 1,2n - 7)$, $r(v_{4,11} |W) = (2n,2n,2n - 6)$, $r(v_{4,12} |W) = (2n + 1,2n + 1,2n - 5)$, $r(v_{4,13} |W) = (2n + 2,2n + 2,2n - 4)$, $r(v_{4,14} |W) = (2n + 3,2n + 3,2n - 3)$, $r(v_{4,15} |W) = (2n + 4,2n + 4,2n - 2)$, $r(v_{4,16} |W) = (2n + 5,2n + 5,2n - 1)$, $r(v_{4,17} |W) = (2n + 5,2n + 6,2n)$, $r(v_{4,18} |W) = (2n + 4,2n + 5,2n + 1)$, $r(v_{4,19} |W) = (2n + 5,2n + 6,2n + 2)$, $r(v_{4,20} |W) = (2n + 4,2n + 5,2n + 3)$, $r(v_{4,21} |W) = (2n + 5,2n + 6,2n + 4)$, $r(v_{4,22} |W) = (2n + 4,2n + 5,2n + 5)$, $r(v_{4,23} |W) = (2n + 5,2n + 6,2n + 6)$, $r(v_{4,24} |W) = (2n + 4,2n + 6,2n + 6)$, $r(v_{4,25} |W) = (2n + 3,2n + 5,2n + 5)$, $r(v_{4,26} |W) = (2n + 2,2n + 4,2n + 6)$, $r(v_{4,27} |W) = (2n + 1,2n + 3,2n + 5)$, $r(v_{4,28} |W) = (2n,2n + 2,2n + 6)$, $r(v_{4,29} |W) = (2n - 1,2n + 1,2n + 5)$, $r(v_{4,30} |W) = (2n - 2,2n,2n + 6)$, $r(v_{4,31} |W) = (2n - 3,2n - 1,2n + 5)$, $r(v_{4,32} |W) = (2n - 4,2n - 2,2n + 4)$, $r(v_{4,33} |W) = (2n - 5,2n - 3,2n + 3)$, $r(v_{4,34} |W) = (2n - 6,2n - 4,2n + 2)$, $r(v_{4,35} |W) = (2n - 7,2n - 5,2n + 1)$.

Representations of vertices on 5th cycle are $r(v_{5,1} |W) = (2n - 10,2n - 8,2n)$, $r(v_{5,2} |W) = (2n - 11,2n - 9,2n - 1)$, $r(v_{5,3} |W) = (2n - 10,2n - 10,2n - 2)$, $r(v_{5,4} |W) = (2n - 9,2n - 9,2n - 3)$, $r(v_{5,5} |W) = (2n - 8,2n - 8,2n - 4)$, $r(v_{5,6} |W) = (2n - 7,2n - 7,2n - 5)$, $r(v_{5,7} |W) = (2n - 6,2n - 6,2n - 6)$, $r(v_{5,8} |W) = (2n - 5,2n - 5,2n - 7)$, $r(v_{5,9} |W) = (2n - 4,2n - 4,2n - 8)$, $r(v_{5,10} |W) = (2n - 3,2n - 3,2n - 9)$, $r(v_{5,11} |W) = (2n - 2,2n - 2,2n - 10)$, $r(v_{5,12} |W) = (2n - 1,2n - 1,2n - 9)$, $r(v_{5,13} |W) = (2n,2n,2n - 8)$, $r(v_{5,14} |W) = (2n + 1,2n + 1,2n - 7)$, $r(v_{5,15} |W) = (2n + 2,2n + 2,2n - 6)$, $r(v_{5,16} |W) = (2n + 3,2n + 3,2n - 5)$, $r(v_{5,17} |W) = (2n + 4,2n + 4,2n - 4)$, $r(v_{5,18} |W) = (2n + 5,2n + 5,2n - 3)$, $r(v_{5,19} |W) = (2n + 6,2n + 6,2n - 2)$, $r(v_{5,20} |W) = (2n + 7,2n + 7,2n - 1)$, $r(v_{5,21} |W) = (2n + 7,2n + 8,2n)$, $r(v_{5,22} |W) = (2n + 6,2n + 7,2n + 1)$, $r(v_{5,23} |W) = (2n + 7,2n + 8,2n + 2)$, $r(v_{5,24} |W) = (2n + 6,2n + 7,2n + 3)$, $r(v_{5,25} |W) = (2n + 7,2n + 8,2n + 4)$, $r(v_{5,26} |W) = (2n + 6,2n + 7,2n + 5)$, $r(v_{5,27} |W) = (2n + 7,2n + 8,2n + 6)$, $r(v_{5,28} |W) = (2n + 6,2n + 7,2n + 7)$, $r(v_{5,29} |W) = (2n + 7,2n + 8,2n + 8)$, $r(v_{5,30} |W) = (2n + 6,2n + 8,2n + 8)$, $r(v_{5,31} |W) = (2n + 5,2n + 7,2n + 7)$, $r(v_{5,32} |W) = (2n + 4,2n + 6,2n + 8)$, $r(v_{5,33} |W) = (2n + 3,2n + 5,2n + 7)$, $r(v_{5,34} |W) = (2n + 2,2n + 4,2n + 8)$, $r(v_{5,35} |W) = (2n + 1,2n + 3,2n + 7)$, $r(v_{5,36} |W) = (2n,2n + 2,2n + 8)$,$r(v_{5,37} |W) = (2n - 1,2n + 1,2n + 7)$, $r(v_{5,38} |W) = (2n - 2,2n,2n + 8)$, $r(v_{5,39} |W) = (2n - 3,2n - 1,2n + 7)$, $r(v_{5,40} |W) = (2n - 4,2n - 2,2n + 6)$, $r(v_{5,41} |W) = (2n - 5,2n - 3,2n + 5)$, $r(v_{5,42} |W) = (2n - 6,2n - 4,2n + 4)$, $r(v_{5,43} |W) = (2n - 7,2n - 5,2n + 3)$, $r(v_{5,44} |W) = (2n - 8,2n - 6,2n + 2)$, $r(v_{5,45} |W) = (2n - 9,2n - 7,2n + 1)$.

So on generalizing we give representations of vertices on $$ith$$ cycle for $$5 < i \le n - 1$$ as $$r(v_{i,1} |W) = (2n - 2i,2n - (2i - 2),2n)$$, $$r(v_{i,2} |W) = (2n - (2i + 1),2n - (2i - 1),2n - 1)$$, $$r(v_{i,3} |W) = (2n - 2i,2n - 2i,2n - 2)$$, $$r(v_{i,4} |W) = (2n - (2i - 1),2n - (2i - 1),2n - 3)$$, $$r(v_{i,5} |W) = (2n - (2i - 2),2n - (2i - 2),2n - 4)$$$$r(v_{i,6} |W) = (2n - (2i - 3),2n - (2i - 3),2n - 5)$$, $$r(v_{i,7} |W) = (2n - (2i - 4),2n - (2i - 4),2n - 6)$$, $$r(v_{i,8} |W) = (2n - (2i - 5),2n - (2i - 5),2n - 7)$$, $$r(v_{i,9} |W) = (2n - (2i - 6),2n - (2i - 6),2n - 8)$$, $$r(v_{i,10} |W) = (2n - (2i - 7),2n - (2i - 7),2n - 9)$$, $$r(v_{i,11} |W) = (2n - (2i - 8),2n - (2i - 8),2n - 10)$$, $$r(v_{i,2i + 1} |W) = v_{i,2i + 1} (2n - 2,2n - 2,2n - 2i)$$, $$r(v_{i,2i + 2} |W) = (2n - 1,2n - 1,2n - (2i - 1))$$, $$r(v_{i,2i + 3} |W) = (2n,2n,2n - (2i - 2))$$, $$r(v_{i,2i + 4} |W) = (2n + 1,2n + 1,2n - (2i - 3))$$, $$r(v_{i,2i + 5} |W) = (2n + 2,2n + 2,2n - (2i - 4))$$, $$r(v_{i,4i} |W) = (2n + 2i - 3,2n + 2i - 3,2n - 1)$$, $$r(v_{i,4i + 1} |W) = (2n + 2i - 3,2n + 2i - 2,2n)$$, $$r(v_{i,4i + 2} |W) = (2n + 2i - 4,2n + 2i - 3,2n + 1)$$, $$r(v_{i,4i + 3} |W) = (2n + 2i - 3,2n + 2i - 2,2n + 2)$$, $$r(v_{i,6i - 1} |W) = (2n + 2i - 3,2n + 2i - 2,2n + 2i - 2)$$, $$r(v_{i,6i} |W) = (2n + 2i - 4,2n + 2i - 2,2n + 2i - 2)$$, $$r(v_{i,6i + 1} |W) = (2n + 2i - 5,2n + 2i - 3,2n + 2i - 3)$$, $$r(v_{i,6i + 2} |W) = (2n + 2i - 6,2n + 2i - 4,2n + 2i - 2)$$, $$r(v_{i,6i + 3} |W) = (2n + 2i - 7,2n + 2i - 5,2n + 2i - 3)$$, $$r(v_{i,8i - 1} |W) = (2n - 3,2n - 1,2n + 2i - 3)$$, $$r(v_{i,8i} |W) = (2n - 4,2n - 2,2n + 2i - 4)$$, $$r(v_{i,8i + 1} |W) = (2n - 5,2n - 3,2n + 2i - 5)$$, $$r(v_{i,10i - 5} |W) = (2n - 2i + 1,2n - 2i + 3,2n + 1)$$.

Representations of vertices on $nth$ cycle are $r(v_{n,1} |W) = (0,2,2n)$, $r(v_{n,2} |W) = (1,1,2n - 1)$ $r(v_{n,3} |W) = (2,0,2n - 2)$, $r(v_{n,4} |W) = (3,1,2n - 3)$, $r(v_{n,2n} |W) = (2n - 1,2n - 3,1)$, $r(v_{n,2n + 1} |W) = (2n,2n - 2,0)$, $r(v_{n,2n + 3} |W) = (2n,2n,2)$, $r(v_{n,2n + 4} |W) = (2n + 1,2n + 1,3)$, $r(v_{n,2n + 5} |W) = (2n + 2,2n + 2,4)$, $r(v_{n,4n} |W) = (4n - 3,4n - 3,2n - 1)$, $r(v_{n,4n + 1} |W) = (4n - 2,4n - 2,2n)$, $r(v_{n,4n + 2} |W) = (4n - 3,4n - 3,2n + 1)$, $r(v_{n,4n + 3} |W) = (4n - 3,4n - 2,2n + 2)$, $r(v_{n,4n + 4} |W) = (4n - 4,4n - 3,2n + 3)$, $r(v_{n,4n + 5} |W) = (4n - 3,4n - 2,2n + 4)$, $r(v_{n,6n - 1} |W) = (4n - 3,4n - 2,4n - 2)$, $r(v_{n,6n} |W) = (4n - 4,4n - 2,4n - 2)$, $r(v_{n,6n + 1} |W) = (4n - 5,4n - 3,4n - 3)$, $r(v_{n,6n + 2} |W) = (4n - 6,4n - 4,4n - 2)$, $r(v_{n,7n - 1} |W) = (3n - 3,3n - 1,4n - 2)$, $r(v_{n,7n} |W) = (3n - 4,3n - 2,4n - 3)$, $r(v_{n,7n + 1} |W) = (3n - 5,3n - 3,4n - 2)$, $r(v_{n,7n + 2} |W) = (3n - 6,3n - 4,4n - 3)$, $r(v_{n,8n} |W) = (2n - 4,2n - 2,4n - 4)$, $r(v_{n,8n + 1} |W) = (2n - 5,2n - 3,4n - 5)$, $r(v_{n,8n + 2} |W) = (2n - 6,2n - 4,4n - 6)$, $r(v_{n,10n - 6} |W) = (2,4,2n + 2)$ and $r(v_{n,10n - 5} |W) = (1,3,2n + 1)$.

These representations are distinct in at least one coordinate. So $W$ is a resolving set for $CNC_{k} [5]$. Hence dim($CNC_{k} [5]$) ≤ 3.

Now we prove that dim($CNC_{k} [5]$) ≥ 3 by proving that any set of cardinality 2 does not resolve it.

### Theorem 2

*For all *$k \ge 1$* we have *$\dim (CNC_{k} [5]) \ge 3$*.*

To prove this we have to prove that no set of vertices with cardinality 2 or less can’t become resolving set for $CNC_{k} [5]$. As $CNC_{k} [5]$ is not a path so possibility of generating set with a single element is ruled out. Now we try for the resolving set with two elements such as $W = \{ v_{p,i} ,v_{q,j} \}$ . We discuss the following possibilities.

### Possibility 1: The vertices in $W$ lie on the same cycle

1. If both vertices in $W$ lie on **first cycle** then $p = q =$ 1 and we have $r(v_{2,2} |W) = r(v_{2,25} |W)$, a contradiction.
2. If both vertices in $W$ lie on **second cycle** then $p = q =$ 2 and we have $r(v_{4,33} |W) = r(v_{4,35} |W)$, a contradiction.
3. If both vertices in $W$ lie on **third cycle** then $p = q =$ 3 and we have $r(v_{6,51} |W) = r(v_{6,53} |W)$, a contradiction.
4. If both vertices in $W$ lie on **fourth cycle** then $p = q =$ 4 and we have $r(v_{8,71} |W) = r(v_{8,73} |W)$, a contradiction.
5. If both vertices in $W$ lie on **fifth cycle** then $p = q =$ 5 and we have $r(v_{10,89} |W) = r(v_{10,91} |W)$, a contradiction.
6. If both vertices in $W$ lie on $rth$ **cycle** and r is odd then $p = q = r$ and we have $r(v_{2r,19r - 6} |W) = r(v_{2r,19r - 4} |W)$, a contradiction.
7. If both vertices in $W$ lie on $(r + 1)th$ **cycle** and r is odd then $p = q = r + 1$ and we have $r(v_{2r + 2,19r + 14} |W) = r(v_{2r,19r + 16} |W)$, a contradiction.
8. If both vertices in $W$ lie on $nth$ **cycle** and then $p = q = n$ and we have $r(v_{n - k,10(n - k) - 5} |W) = r(v_{n - k - 1,2} |W)$ for $k = 0$ or $k = 1$ or $k = 2$, or ……, or $k = n{-}2$.

### Possibility 2: The vertices in $W$ lie on two different neighboring cycles

1. If one vertex in $W$ lies on first cycle $C_{1}$ and the second vertex in $W$ lies on second cycle $C_{2}$. Without loss of generality we suppose that the vertex on $C_{1}$ is $v_{1,1}$ and the vertex on $C_{2}$ is $v_{2,j}$; $1 \le j \le 15$. Then $v_{3,2} = v_{3,25}$ or $v_{3,2} = v_{4,35}$, a contradiction.
2. If one vertex in $W$ lies on $C_{2}$ and the second vertex in $W$ lies on $C_{3}$. Without loss of generality we suppose that the vertex on $C_{2}$ is $v_{21}$ and the vertex on $C_{3}$ is $v_{3,j}$; $1 \le j \le 25$. Then $v_{4,2} = v_{4,35}$ or $v_{4,2} = v_{5,45}$, a contradiction.
3. If one vertex in $W$ lies on $C_{3}$ and the second vertex in $W$ lies on $C_{4}$. Without loss of generality we suppose that the vertex on $C_{3}$ is $v_{3,1}$ and the vertex on $C_{4}$ is $v_{4,j}$; $1 \le j \le 35$. Then $v_{5,2} = v_{5,45}$ or $v_{5,2} = v_{6,55}$, a contradiction.
4. If one vertex in $W$ lies on $C_{i}$ and the second vertex in $W$ lies on $C_{i + 1}$ where $i$ is odd. Without loss of generality we suppose that the vertex on $C_{i}$ is $v_{i,1}$ and the vertex on $C_{i + 1}$ is $v_{i + 1,j}$; $1 \le j \le 10i + 5$. Then $v_{i + 2,2} = v_{i + 2,i + 15}$ or $v_{i + 2,2} = v_{i + 3,i + 25}$, a contradiction.
5. If one vertex in $W$ lies on $C_{n - 1}$ and the second vertex in $W$ lies on $C_{n}$. Without loss of generality we suppose that the vertex on $C_{n}$ is $v_{n,1}$ and the vertex on $C_{n - 1}$ is $v_{n - 1,j}$; $1 \le j \le 10n - 15$. Then $v_{n,2} = v_{n,10n - 5}$ or $v_{n - 1,2} = v_{n,10n - 5}$, a contradiction.

### Possibility 3: The vertices in $W$ lie on two different cycles which are not neighboring

1. If one of the vertices in $W$ lie on $C_{1}$ and the other vertex lie on $C_{i}$ where $3 \le i \le n{-}2$. Without loss of generality suppose the vertex on $C_{1}$ is $v_{1,1}$ and the vertex on $C_{i}$ is $v_{i,j}$ where $1 \le j \le 10i - 5$. Then $v_{i + 1,2} = v_{i + 1,10i + 5}$ or $v_{i + 2,1} = v_{i + 1,3}$, a contradiction.
2. If one of the vertices in $W$ lie on $C_{1}$ and other vertex lie on $C_{n - 1}$. Without loss of generality suppose the vertex on $C_{1}$ is $v_{1,1}$ and the vertex on $C_{n - 1}$ is $v_{n - 1,j}$ where $1 \le j \le 10n - 15$. Then $v_{1,2} = v_{1,5}$ or $v_{1,2} = v_{2,1}$, a contradiction.
3. If one of the vertices in $W$ lie on $C_{1}$ and other vertex lie on $C_{n}$. Without loss of generality suppose the vertex on $C_{1}$ is $v_{1,1}$ and the vertex on $C_{n}$ is $v_{n,j}$ where $1 \le j \le 10n - 5$. Then $v_{1,2} = v_{1,5}$ or $v_{1,2} = v_{2,1}$, a contradiction.
4. If one of the vertices in $W$ lie on $C_{i}$ and other vertex lie on $C_{k}$ where $1 < i < k < n$. Without loss of generality suppose the vertex on $C_{i}$ is $v_{i,1}$ and the vertex on $C_{k}$ is $v_{k,j}$ where $1 \le j \le 10k - 5$. Then $v_{i - 1,1} = v_{i,10i - 5}$ or $v_{i - 1,1} = v_{i,2}$, a contradiction.

From above discussion it is concluded that any set of cardinality two does not resolve $CNC_{k} [5]$. So dim($CNC_{k} [5]$) > 2. Hence using two main results of this section, we finally arrive at, dim($CNC_{k} [5]$) = 3.

## Conclusions and discussions

Resolving sets for a particular network or a graph carry important information essential for the unique identification of each component present in the network. We prove that 1-pentagonal nanocone network, $CNC_{k} [5]$**,** represents a family of bounded, constant metric dimension 3 and it doesn’t depend upon the value of $k$. Moreover, we also give a particular class of metric basis and metric generators for $CNC_{k} [5]$. Results established in this article can be useful to people working in the area of Nano-engineering, nano-devices and micro-devised constructed from $CNC_{k} [5]$. In nano-biotechnology and pharmacy, these results can also be helpful in drug designs where different nodes are of different significance. We also pose natural open problem of extending these results to other subfamilies of 1-hexagonal, 1-heptagonal and all other carbon nanocones.
